# Changes in retinal layer thickness with maturation in the dog: an in vivo spectral domain - optical coherence tomography imaging study

**DOI:** 10.1186/s12917-020-02390-8

**Published:** 2020-06-30

**Authors:** Laurence M. Occelli, Nate Pasmanter, Elias E. Ayoub, Simon M. Petersen-Jones

**Affiliations:** Department of Small Animal Clinical Sciences, College of Veterinary Medicine, Michigan State University, 736 Wilson Road, D-208, East Lansing, MI 48824 USA

**Keywords:** Dog, Retina, Maturation, SD-OCT, *Area centralis*

## Abstract

**Background:**

Retinal diseases are common in dogs. Some hereditary retinal dystrophies in dogs are important not only because they lead to vision loss but also because they show strong similarities to the orthologous human conditions. Advances in in vivo non-invasive retinal imaging allow the capture of retinal cross-section images that parallel low power microscopic examination of histological sections. Spectral domain - optical coherence tomography (SD-OCT) allows the measurement of retinal layer thicknesses and gives the opportunity for repeat examination to investigate changes in thicknesses in health (such as changes with maturation and age) and disease (following the course of retinal degenerative conditions). The purpose of this study was to use SD-OCT to measure retinal layer thicknesses in the dog during retinal maturation and over the first year of life. SD-OCT was performed on normal beagle cross dogs from 4 weeks of age to 52 weeks of age. To assess changes in layer thickness with age, measurements were taken from fixed regions in each of the 4 quadrants and the *area centralis* (the region important for most detailed vision). Additionally, changes in retinal layer thickness along vertical and horizontal planes passing through the optic nerve head were assessed.

**Results:**

In the four quadrants an initial thinning of retinal layers occurred over the first 12 to 15 weeks of life after which there was little change in thickness. However, in the *area centralis* there was a thickening of the photoreceptor layer over this time period which was mostly due to a lengthening of the photoreceptor inner/outer segment layer. The retina thinned with greater distances from the optic nerve head in both vertical and horizontal planes with the dorsal retina being thicker than the ventral retina. Most of the change in thickness with distance from the optic nerve head was due to difference in thickness of the inner retinal layers. The outer retinal layers remained more constant in thickness, particularly in the horizontal plane and dorsal to the optic nerve head.

**Conclusions:**

These measurements will provide normative data for future studies.

## Background

The canine retina is immature at birth and completes development and maturation over the first few weeks of age. The histological and functional changes during this period have been previously described [1–4]. The inner layer of the optic cup proliferates and differentiates into inner and outer neuroblastic layers which differentiate into the various inner and outer retinal neurons and glia. Retinal ganglion cells develop first, while the cone photoreceptor cells develop later followed by bipolar and rod photoreceptor cells which mature last. Photoreceptor cell development occurs in a centrifugal central to peripheral fashion, with inner segments first extending through the outer limiting membrane in late gestation or early postnatally [4]. They achieve adult conformation centrally at day 15 and by day 19 peripherally [4]. Outer segments develop from the cilium of the inner segments and this also occurs in a central to peripheral fashion. The photoreceptors appear to be fully developed centrally at about 6 weeks of age and peripherally by 10 weeks of age [2].

Spectral domain – optical coherence tomography (SD-OCT) is a non-invasive method that can be used to obtain high resolution cross-section images of the retina. Recent advances mean that commercial instruments have an axial resolution of about 3.5 μm/pixel digital (7 μm optical) [5, 6] allowing images that resemble low power histological retinal cross-sections to be obtained. The histological origin of the different reflective bands imaged from the retina is mostly understood and changes in these layers with disease have been investigated [7–9]. The technique can be used for longitudinal assessment of the retina in individual animals (or people) allowing for physiological and pathological changes in layer thicknesses and morphology to be monitored. SD-OCT has already been used in dogs to establish normal parameters [10–15], investigate the phenotype of a number of retinal conditions [16–24], and also the effect of therapies on retinal structure [21, 25–27].

During retinal maturation, following cessation of active cell division, the globe increases in size meaning that the surface area of the retina increases thus spreading the same tissue over a greater surface area [28]. Similar to other neural tissue, there is some loss of neurons in the maturing retina due to apoptosis [29]. This is a method to remove neurons that have failed to make the correct connections and is reported in all layers of the developing human retina [30] as well as in other species including the dog [31, 32]. These factors mean that there are likely changes in retinal layer thicknesses as the eye matures resulting from both retinal ‘stretch’ and also loss of neurons. Normal changes of retinal layer thickness may complicate interpretation of retinal layer thickness changes in dogs with early-onset retinal dystrophies and also in judging the effect of therapy in such animals.

The purpose of the current study is to investigate the longitudinal changes in retinal thickness in the four retinal quadrants as well as the *area centralis* in normal dogs during retinal maturation.

## Results

### Changes in retinal thickness with age in the four quadrants and *area centralis*

**Fig. 1:**
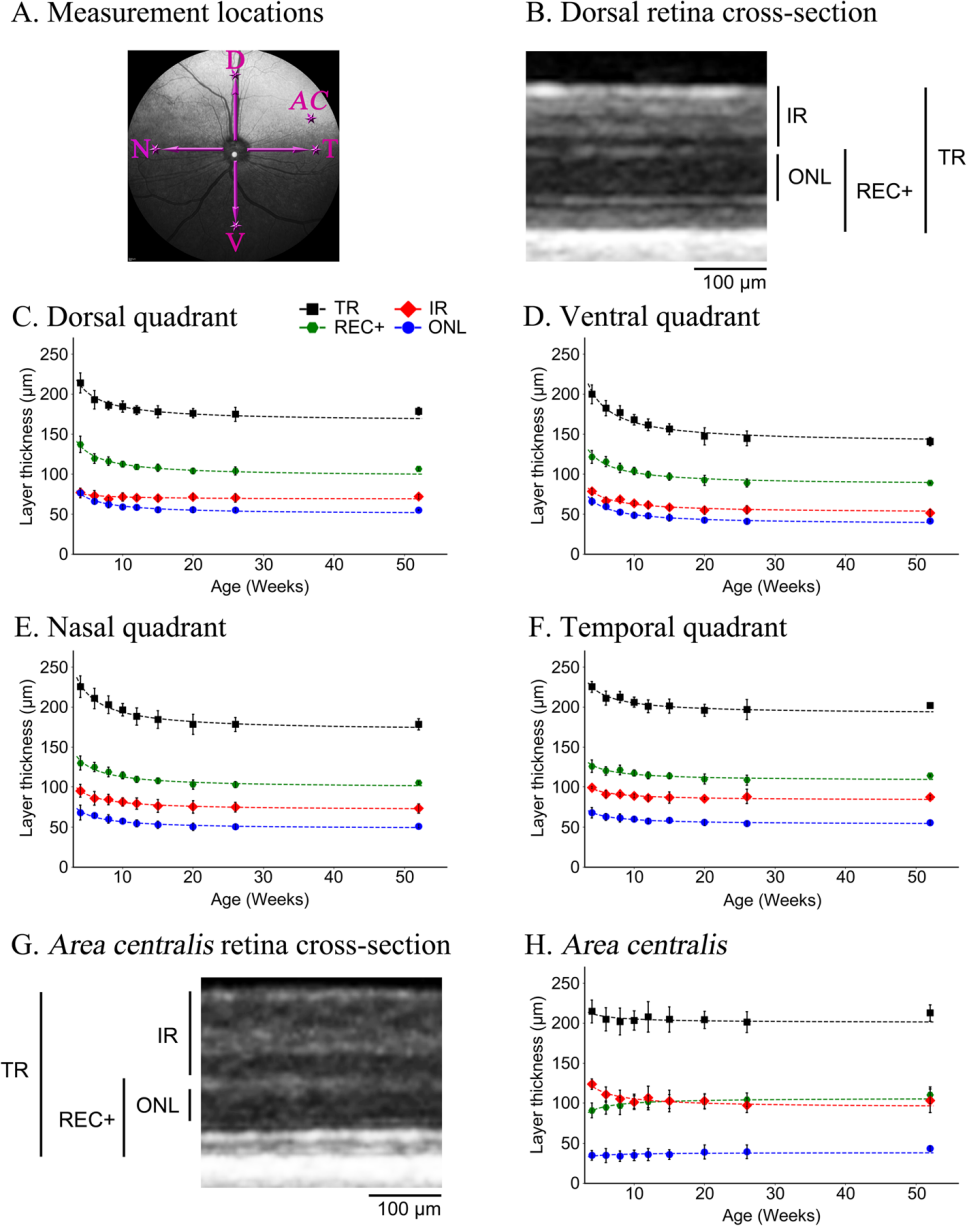
Changes in retinal layer thicknesses with age in the 4 regional quadrants and *area centralis.***a**. Confocal scanning laser ophthalmoscopy (cSLO) image of a left eye fundus indicating the sites at which retinal layer thicknesses were measured. **b**. Representative SD-OCT high resolution cross-section image of the retina in the dorsal quadrant in a 12-week-old dog with adjacent bars showing the different layers measured: total retina (TR), Receptor+ (REC+), inner retina (IR), and outer nuclear layer (ONL). **c-f**. The mean (+/− SD) thicknesses of the measured retinal layers in the dorsal (**c**), ventral (**d**), nasal (**e**) and temporal (**f**) quadrants with age. Each dataset is fitted with a linear regression model. All layers in each of the regions thinned between 4 and 12 weeks of age. A repeated measures correlation showed there was a significant linear correlation between decrease in thickness of each of the four layers in each of the regions and age. See Additional file 1 - Tables S2A and S2B for r and *p*-values, respectively, as well as Additional file 1 - Tables S1A and S1B for raw values and percentage changes with age respectively. **g**. Representative SD-OCT high resolution cross-section images of the retina in the *area centralis* in a 12-week-old dog with adjacent bars showing the different layers measured: total retina (TR), Receptor+ (REC+), inner retina (IR), and outer nuclear layer (ONL). Note the thicker IR layer in the *area centralis* region compared to the IR thickness in the dorsal region (**b**). **h**. The mean (+/− SD) thicknesses of the measured retinal layers in the *area centralis* with age. Each dataset is fitted with a linear regression model. IR thinned to about 8 to 10 weeks of age while the REC+ thickened over this period and the ONL and TR showed little change in thickness with age. See Additional file 1 - Tables S1A and S1B for raw values and percentage changes with age respectively, as well as Additional file 1 - Tables S2A and S2B for r and *p*-values, respectively

**Fig. 2:**
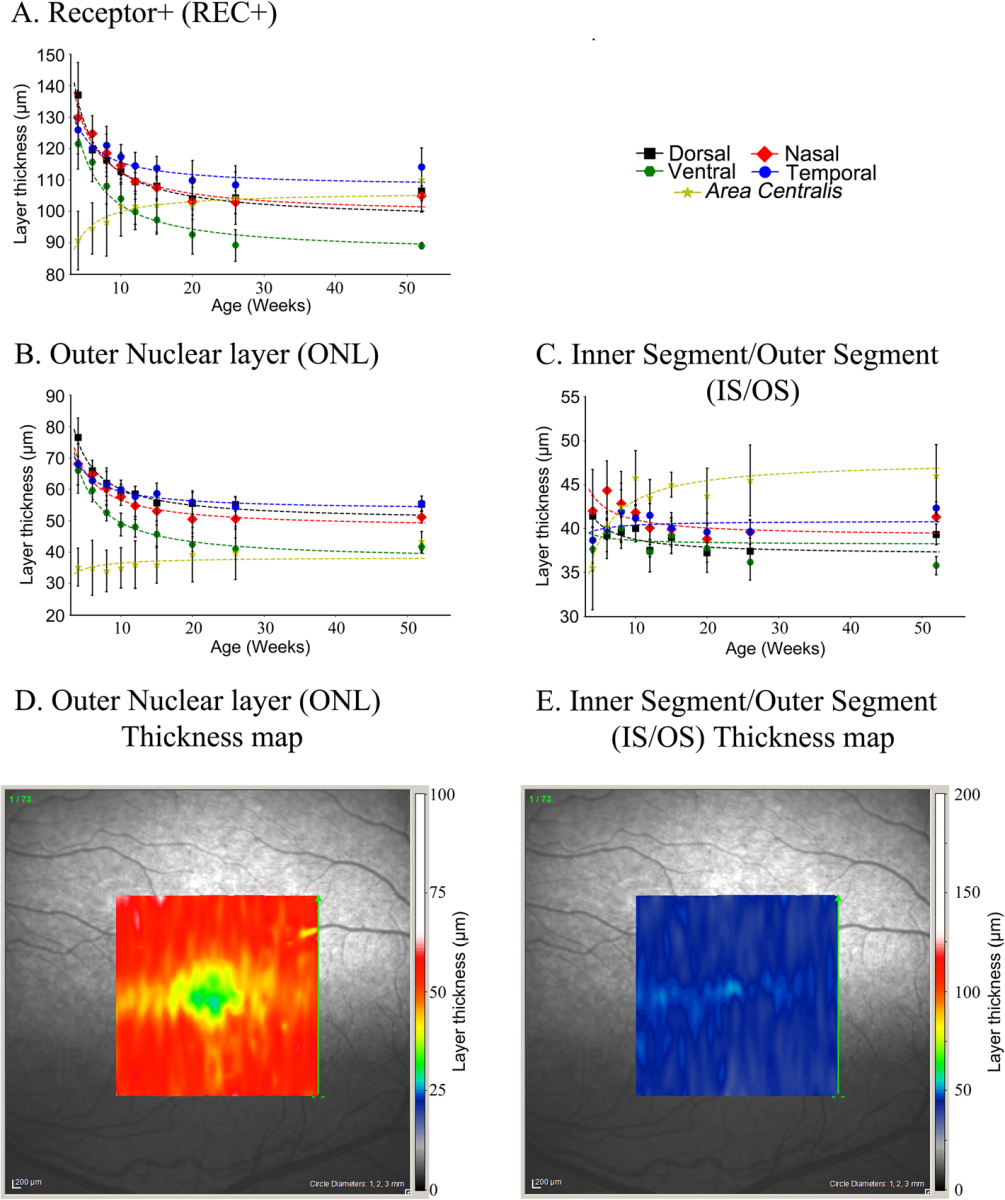
Mean layer thicknesses with age of REC+, ONL, IS/OS and heat map in the *AC.***a-c**. Mean (+/− SD) layer thicknesses with age of **a**. Receptor+ (REC+), **b**. Outer nuclear layer (ONL), **c**. Photoreceptor inner segment/outer segment (IS/OS) for the 5 regions. Note that in the dorsal, ventral, nasal and temporal regions the REC+ thinned with age. In the *area centralis* it was thinner than in the other regions and trended towards thickening with age (A). The ONL in the *area centralis* changed little with age (whereas in the other areas in thinned). The change in REC+ thickness with age in the *area centralis* seemed to be accounted for primarily by thickening of the IS/OS. **d**, **e**. Heat maps showing layer thickness of ONL and IS/OS in the region of and surrounding the *area centralis* in a 12-week-old dog. This shows the regionality of the difference in thickness of these 2 layers

SD-OCT high-resolution retinal cross-section images were obtained from each of the 4 retinal quadrants and the *area centralis* from the same 4 dogs from 4 to 52 weeks of age. For the quadrants, the measurements were taken 4 optic nerve head widths from the edge of optic neural canal rim (optic rim) in the superior, inferior, nasal and temporal directions (Fig. 1a). The same location was measured in each dog at each age with the aid of the automatic follow up scan placement software (AutoRescan Heidelberg Engineering, Heidelberg, Germany). The mean measurements for all retinal layers measured at each of the 5 regions at each timepoint are shown in Additional file 1 - Table S1A. The percentage changes in thicknesses compared to the layer thicknesses at 4 weeks of age are also shown in Additional file 1- Table S1B. The changes in thickness of total retina (TR), inner retina (IR), Receptor+ (REC+) and outer nuclear layer (ONL) in each of these regions over the first year of age are shown graphically in Figs. 1c-f. Changes in the inner nuclear layer (INL) and ganglion cell complex (GCC: layers between inner plexiform layer and internal limiting membrane) layer thicknesses are shown in Additional file 2 - Figure S1.

(i.) **Changes in the 4 quadrants:** There were similar changes at each of the 4 quadrants; from 4 to approximately 12–15 weeks of age there was a decrease in thickness of TR, IR, REC+ and ONL after which there was little further change in thickness up to 52 weeks of age. A linear regression fit these data well and showed a significant negative correlation of thickness with age (Additional file 1 - Tables S2A and S2B show the r and *p*-values for the correlations, respectively). Comparing the 4 quadrants, the ventral quadrant showed the most pronounced thinning of the layers over the first 12 weeks of age followed by the nasal, dorsal then temporal quadrants (Figs. 2a-c, Additional file 2 - Fig. S1 and Additional file 1 - Tables S1A and SB). For example, the REC+ thickness of the ventral quadrant decreased to 73.3% of the thickness at 4 weeks of age by 52 weeks of age while it decreased to 77.7, 80.8 and 90.6% for the dorsal, nasal and temporal quadrants respectively. The IR thickness of the ventral quadrant decreased to 65.7% of the thickness at 4 weeks of age by 52 weeks of age while it decreased to 93.6, 76.9 and 88.2% for the dorsal, nasal and temporal quadrants respectively. After 15 weeks of age there was little change in layer thicknesses.
(ii.) **Changes in the*area centralis*:** Changes in the inner retinal layer thicknesses with age showed similar trends in the *area centralis* to the 4 quadrants. However changes in the REC+ with age differed from that in the 4 quadrants, in the *area centralis* it thickened over the first few weeks of age (Figs. 1g-h, 2a) while over this time period in the 4 quadrants it thinned. To further investigate this difference between the *area centralis* and the 4 quadrants we looked more closely at the components that make up the REC+. The REC+ represents the entire length of the photoreceptor from the outer plexiform layer to the retinal pigment epithelium and includes the ONL and the length of the photoreceptor inner and outer segments (IS/OS). These are shown separately (Fig. 2) and per region (Additional file 3 - Fig. S2). The ONL (representing the photoreceptor nuclear layer) in the *area centralis* changed little in thickness with age (Figs. 1h and 2b) while it thinned in the 4 quadrants. The thickness of the layers representing the IS/OS (Fig. 2c and Additional file 3 - Fig. S2) increased in thickness in the *area centralis* over the first 12 to 15 weeks accounting for the change measured in the REC+ in this region. In comparison, the IS/OS layer thickness in the 4 quadrants remained similar or slightly decreased (Fig. 2c and Additional file 3 - Fig. S2). The topography of the REC+ and IS/OS thickness in the *area centralis* and surrounding retina are shown by the heat maps in Fig. 2d and e at 12 weeks of age. These illustrate that the ONL around the center of the *area centralis* is thinner than the surrounding retina whereas the IS/OS layer is thicker at the very center of the *area centralis*.

### Changes in retinal layer thickness in the vertical and horizontal planes

**Fig. 3:**
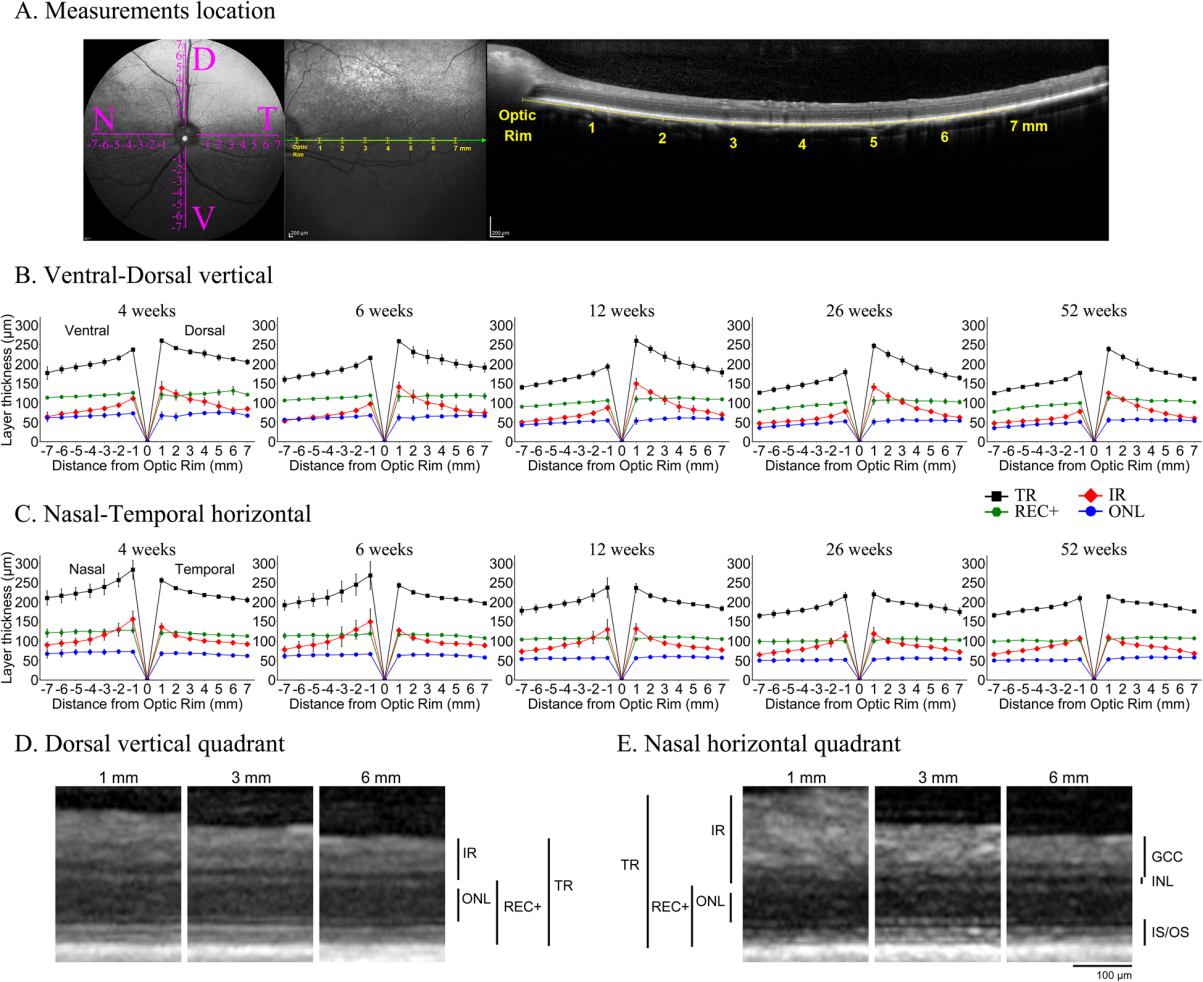
Retinal layer thicknesses changes with age and eccentricity in the dorso-ventral and naso-temporal planes. **a** cSLO fundus image showing planes along which retinal layer thickness were measured. The temporal plane is shown in more detail with the corresponding SD-OCT image showing positions where measurements were made. **b** and **c** Spider graphs showing the mean (+/− SD) total retina (TR), inner retina (IR), Receptor+ (REC+) and outer nuclear layer (ONL) thicknesses in the ventro-dorsal (**b**) and naso-temporal (**c**) axes at 4, 6, 12, 26 and 52 weeks of age. Note that at each age the total retina (TR – shown in black in the figures) thins with distance from the optic rim. This is mostly accounted for by thinning of the inner retina (IR, red). The outer retina (REC+ and ONL) shows little change with distance from the optic rim (also see Fig. 4). The dorsal retina is thicker than the ventral retina at all ages and the nasal retina slightly thicker than the temporal retina at early ages (4 and 6 weeks of age). A linear mixed effects model was calculated to analyze layer thickness changes with increasing distance from the optic rim. Correlation of layer thickness with distance from optic rim and significance values are shown in Additional file 1 - Tables S4A and 4B, respectively. Additional file 1 - Tables S3A and S3B show raw values and percentage changes with eccentricity, respectively. **d**-**e** Representative SD-OCT high resolution cross-section images of the retina at 12 weeks of age of the dorsal (**d** oriented vertically**)** and nasal (**e** oriented horizontally**)** quadrants centered at 1, 3 and 6 mm from the edge of the optic rim. The adjacent bars show the measurement of the different retinal layers. Note the progressive thinning of the TR and IR from 1 to 6 mm from the optic rim while the REC+ and ONL thicknesses show little change

**Fig. 4:**
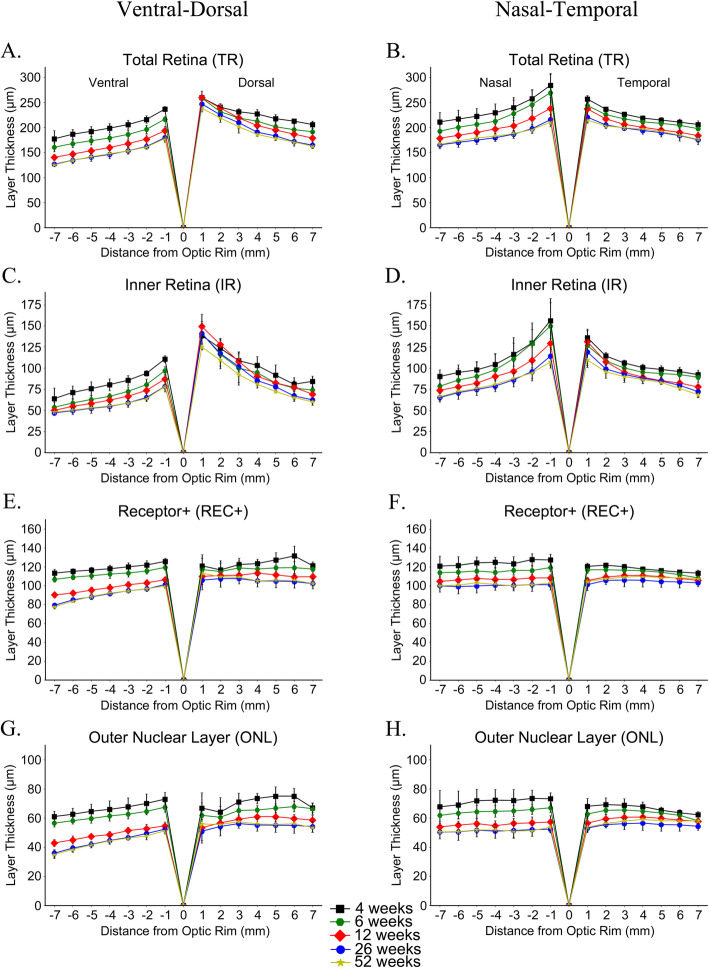
Spider graphs showing the mean of each retinal layer thicknesses with eccentricity and age. Mean (+/− SD) of retinal layer thicknesses in the ventro-dorsal (**a**, **c**, **e** and **g**) and naso-temporal (**b**, **d**, **f** and **h**) axes from 1 to 7 mm from the optic rim at 4, 6, 12, 26 and 52 weeks of age. Total retina (TR; **a** and **b**), inner retina (IR; **c** and **d**), Receptor+ (REC+; **e** and **f**), outer nuclear layer (ONL; **g** and **h**) and are shown. The total retina thins with distance from optic nerve rim in all directions (**a** and **b**) mostly accounted for by thinning of the inner retina (**c** and **d**). The outer retina (ONL and REC+) thins with distance from the optic rim ventrally (**e** and **g**) while dorsally (**e** and **g**) and in the naso-temporal plane (**f** and **h**) there is little thinning with distance from the optic rim

SD-OCT high resolution retinal cross-section images were also acquired along a vertical and horizontal plane through the optic nerve from each of the 4 dogs at 5 separate timepoints (4, 6, 12, 26, and 52 weeks of age) (Fig. 3a, d and e). Retinal layer thicknesses at 1 mm increments from 1 to 7 mm from the optic rim were measured and “spider graphs” created (Fig. 3b and c). Because of growth in the size of the eye 7 mm from the edge of the optic rim at 52 weeks of age will not be the exact equivalent retinal region to 7 mm from the edge of the optic rim at 4 weeks of age. However, these measurements provide a normal reference dataset for retinal layer thicknesses at each age and provide a further insight into regional differences in retinal layer thickness and changes with age. The mean measurements at each point and age are shown in Additional file 1 - Table S3A and the percentage of retinal layer thicknesses changes relative to the value at 1 mm distance from the optic rim are displayed in Additional file 1 - Table S3B. Figure 3 shows the TR, IR, REC+ and ONL for the vertical and horizontal planes through the optic nerve for each age separately. Figure 4 displays the same measurements for the two planes with each layer separately to better allow comparison of thicknesses with age. Additional file 4 - Figure S3 shows the measurements of the inner nuclear layer (INL), ganglion cell complex (GCC) and inner/outer photoreceptor segments (IS/OS). A mixed linear model was performed on each area with respect to distance from the optic rim for TR, REC+, ONL, IR, INL, GCC, and IS/OS layer thicknesses (see Additional file 1 - Tables S4A and S4B for r and *p*-values, respectively). TR became thinner with increasing distance from the optic rim to the periphery in all four eccentricities and at all ages (Figs. 3b, c, 4a and b). This was predominantly accounted for by thinning of the IR with distance from the optic rim (Figs. 3b, c, 4c and d). The outer retina (REC+ and ONL) thinned with progressive distance from the optic rim in the ventral plane but changed little in thickness with distance from the optic rim dorsally, nasally and temporally (Figs. 3b, c and 4e-h). Additionally, while all layers in these regions thinned to a certain extent with age, the relative trend of thickness changes with increasing distance from the optic rim remained consistent at all measured timepoints for the TR, REC+, ONL, and IR (Figs. 3 and 4) as well as the INL, GCC, and IS/OS (Additional file 4 - Figure S3). Figure 4 and Additional file 1 - Table S3A and 3B show the trend in changes of layer thickness with age. There was thinning in most retinal layers between 4 and 6, 6 and 12 and 12 and 26 weeks of age with very little difference in retinal layer thicknesses between the 26 and 52 week timepoints. The INL showed noticeable thinning between 4 and 12 weeks of age whereas the GCC and overall IR did not thin to such a degree (Additional file 4 - Fig. S3 and Fig. 4). Considering the outer retina there was little change with age in the combined IS/OS layer width with age while the ONL thinned with age, showing the most thinning between 6 and 12 weeks of age.

## Discussion

Spectral domain – optical coherence tomography allows the collection of high resolution retinal cross-section images in a non-invasive fashion. As the technique has become further refined images that are comparable to low power histological sections of the retina can be obtained. Studies have shown the correlation between the SD-OCT images, which are generated from the reflected laser light, and the structural components of the retina as seen on histology [7, 8, 33–36]. SD-OCT allows for the investigation of retinal changes and even the detection of subtle changes in retinal morphology that may not be detectable by funduscopic examination. This is incredibly valuable for the detailed assessment of the retina in the living animal. Alterations of the retina that result from disease processes can be detected and followed longitudinally as can normal physiological changes.

As the retina and eye matures it is known that morphological changes occur in the retina and that the globe increases in size. The current study provides a detailed set of measurements of the different layers of the canine retina discernible by SD-OCT and shows how they change in thickness over the period of retinal maturation from 4 weeks of age, with most change occurring before 15 weeks of age. Repeated measurement from 4 retinal regions (dorsal, ventral, temporal and nasal to the optic nerve head) showed that there was thinning of all retinal layers measured between 4 weeks and 12 to 15 weeks of age after which there was little change in layer thicknesses. The *area centralis* of the dog is detectable as a region of higher photoreceptor density dorsotemporal to the optic nerve head [15, 37]. On SD-OCT imaging it can be localized by a very localized thinning of the outer nuclear layer (ONL) as well as thickening of the INL and GCL as seen in Fig. 1g. In contrast to the situation in the four quadrants where the REC+ thinned with retinal maturation, in the *area centralis* it thickened (Fig. 2a). The REC+ represents the entire length of the photoreceptor and on SD-OCT measurement includes the ONL, the OPL (outer plexiform layer) and the photoreceptor inner/outer segments (IS/OS) combined layers. Investigating each of these layers showed that most of the thickening of the REC+ in the *area centralis* was accounted for by increased thickness of the IS/OS layer (Fig. 2c). As the *area centralis* matures it appears that there is an elongation of the combined length of the inner and outer photoreceptor segments so that the IS/OS layer in the *area centralis* becomes is thicker than in the four quadrants. The tighter packing density of photoreceptors in the *area centralis* means that in this region the photoreceptor inner and outer segments are long and thin.

The general trend of thinning of the retina over the period of retinal maturation may result from a combination of the pruning of neurons that fail to make the appropriate connections [30] and the effect of retinal spread as the globe enlarges and the surface are of the retina (which is at this stage post-mitotic) increases [28]. The changes of retinal layer thicknesses with age closely follow the changes in axial length, where most rapid changes occur between 2 and 9 weeks of age with a plateau being reached at about 20 weeks of age [28].

Factoring in the normal physiological thinning of the retina that occurs with maturation is important when evaluating dogs with an early-onset retinal degeneration, so that normal physiological thinning can be allowed for when assessing the degree of disease-related thinning of retinal layers. This is also of importance when assessing the outcome of therapeutic interventions for retinal degenerative conditions (such as gene augmentation therapy for treating early-onset conditions [25]) so as not to confuse the normal physiological thinning that occurs with a failure of the therapy to halt progression of retinal degeneration.

To provide additional information about retinal layer thickness differences with eccentricity, we also measured thicknesses every mm from the optic rim dorsally, ventrally, temporally and nasally up to 7 mm from the optic rim. This showed significant thinning of the inner retinal layers with distance from the optic rim in addition to a slight thinning of the outer retina ventrally, but demonstrated little thickness change of the outer retina dorsally, temporally, and nasally. In fact, the IS/OS thickness changed little with eccentricity (note that the nasal-temporal plane did not go through the *area centralis*). A recent publication in female beagles reported the SD-OCT measured thickness of total retina, outer retina and nerve fiber layer of a range of ages in similar planes to those we used [14]. Similarly to our study, they found that the retina was thickest in puppies and that the retinal layers thinned progressively with increased distance from the optic nerve head. They reported that the greatest degree of thinning with eccentricity was in the nerve fiber layer. While we did not specifically measure the nerve fiber layer it is included in the inner retina component reported in our study. Measurement of the peripheral retinal thickness in humans with SD-OCT shows similar findings to those that we report here with increasing thinning with distance from the central retina [38]. Wenner et al. [38] also reported that peripheral retinal thickness in young adults was significantly higher in the nasal rather than temporal retina. We did not see the same trend in dogs but did note that the ventral retina tended to be thinner than other areas.

The data presented here provides a normative baseline of layer thicknesses with age and retinal location. There are likely to be breed differences, so this dataset may only be closely applicable for studies in the laboratory beagle. Dogs do show breed differences in ganglion cell density within the visual streak and *area centralis* with differences being correlated with skull shape [39]; brachycephalic breeds having a higher ratio of peak numbers of ganglion cells in the *area centralis* to the visual streak than dolichocephalic breeds. In this study, we only investigated laboratory mix-beagles and it is conceivable that the retinal layer thicknesses may differ in other breeds and perhaps also be related to cephalic index. Investigation of breed variability in SD-OCT measured retinal layers would be a valuable further study. The inclusion of only 2 animals of each sex may not be sufficient to show differences in thickness between male and female dogs. In humans a definite difference between the sexes in the thickness of different retinal layers is reported [40]. Further studies to investigate difference between sexes in the dog are also needed. Additionally, during maturation the axial length of the globe changes which potentially may introduce some inaccuracy in the measurements between different ages. However more significant effects are likely to be due to difference in magnification of the SD-OCT image of retinal layers between eyes with different refractive errors [41]. Fortunately most normal dogs only have minor refractive errors but the effect of changes in refractive state with maturation of puppies could be considered in future studies.

## Conclusion

We show that there is a general thinning of most retinal layers as the dog matures. There is also a negative correlation of thickness of most retinal layers with increasing eccentricity, and the ventral retina tended to be thinner than the retina in other planes. In the *area centralis*, lengthening of the combined IS/OS occurred and may be related to the higher packing density of photoreceptors in this region. This paper and the supplemental data sets provide a detailed baseline of retinal layer thicknesses in the beagle cross during maturation.

## Methods

### Ethics statement

All procedures were performed in accordance with the ARVO statement for the Use of Animals in Ophthalmic and Vision Research and approved by the Michigan State University Institutional Animal Care and Use Committee.

### Animals

Four normal beagle-cross dogs from a colony of dogs maintained at Michigan State University were used in this study. The 4 animals (2 males, 2 females) were from a breeding colony used in other unrelated studies. They were housed under 12 h:12 h light: dark cycles and fed a commercial complete dog diet. Each animal underwent fundus imaging at 4, 6, 8, 10, 12, 15, 20, 25–26 and 52 weeks of age. The dogs were then returned to the breeding colony for other unrelated studies.

Routine ophthalmoscopic examinations were performed prior to each imaging session to confirm that there were no ocular abnormalities.

### Anesthesia

Imaging was performed under general anesthesia. Animals under 15 weeks of age were induced with isoflurance delivered by mask and then intubated and maintained with isoflurane delivered in oxygen. Those over 15 weeks of age were premedicated with subcutaneous acepromazine (0.05–0.2 mg/kg, Henry Schein Animal Health). Anesthesia was induced with intravenous propofol (4 to 6 mg/kg, PropoFlo, Abbott Animal Health), intubated and anesthesia maintained with isoflurane (Isoflo, Abbott Laboratories, between 2 and 3.5% in a 1–2 L/min oxygen flow) via a rebreathing circle system for dogs over 10 kg and via a Bain system for dogs under 10 kg.

### Collection of retinal cross-sectional images

High definition retinal cross-section images (single line scan and volume scans) were collected using the Spectralis OCT + HRA (Heidelberg Engineering). To allow for the differing magnification between eyes of different sizes corneal curvature was factored in based on previous publications [42].

To allow for a direct comparison of retinal layer thickness measurements with age, images were captured from the same 5 retinal regions at each age; the *area centralis* (identified by the focal thickening of the ganglion cell layer in this region), the superior, inferior, nasal and temporal fundus at 4 optic nerve head widths from the edge of the optic nerve on the initial examination (Fig. 3a) then the AutoRescan software (Heidelberg Engineering) was used to follow-up the same location at the following timepoints. This automatic follow up scan placement software with minor manual adjustments was used to ensure the same precise regions were imaged at each age. If the AutoRescan could not identify the previously imaged region landmarks using vessels location was used to find the same location of measurements in the best of our abilities. To provide normative baseline thicknesses for use in future studies vertical and horizontal retinal cross-sectional images were recorded through the optic nerve head. These latter images were also used to measure retinal layer thickness every millimeter (up to 7 mm) from the edge of the optic nerve head. Total retinal (TR), Receptor+ (REC+; including layers between retinal pigmentary epithelium and outer plexiform layer), outer nuclear layer (ONL), inner nuclear layer (INL), ganglion cell complex (GCC: layers between inner plexiform layer and internal limiting membrane) and inner retina (IR: layers between inner nuclear layer and internal limiting membrane) thicknesses were measured using the Heidelberg Eye Explorer (HEYEX) software (Heidelberg Engineering) (Figs. 1a and b, 2d and e, 3a, d and e, and Additional file 3 - Fig. S2E). Areas with major retinal blood vessels were avoided. Three measures were performed in the superior, inferior, nasal and temporal areas and averaged for each area and timepoints except for the center of the *area centralis* which only one measure was recorded for each timepoints. The averaged superior, inferior, nasal and temporal areas (not including *area centralis*) are collectively referred to as peripheral area/region in this paper even though they represent measurements in the central retina. *Area centralis* represents in the region of high rods/cones density as indicated in the introduction.

### Statistical analysis

A repeated measures correlation designed by Bakdash et al. [43] was used to determine if a relationship existed between age and the measures in this study. This method utilizes a variation of ANCOVA, accounting for the effect of individual variability on measurements. The output of this technique, rmcorr, represents the strength of the correlation between dependent and independent variables, with statistical significance determined by the F-test. A mixed effect model using statsmodels in the Python interface was used to analyze the single millimeter eccentricity measurements for SD-OCT evaluated over time. The equation below was used ${Y}_i={\sum}_{i=0}^n\beta \mathrm{X}+{\alpha}_i+{\varepsilon}_{i.}$

Where β is the parameter vector, X is the independent variable matrix, *α*_*i*_ is the dog level residual, and the *ε*_*i*_ is the individual observation level residual [44, 45].

## Supplementary information

**Additional file 1 Table S1A**. Raw retinal values layer thicknesses (in μm) for each age at 4 optic nerve distance from the optic disk rim. **Table S1B.** Percentage of retinal layer thicknesses changes relative to the value at 4 weeks of age (raw values in Table S1A). **Table S2A.** Correlation r values between a given retinal layer thicknesses and retinal region with age. **Table S2B.***P*-values for correlation r values corresponding to a given retinal layer thickness change and retinal region with age and area (corresponding to r value in Table S2A). *P*-values for a given correlation coefficient are calculated via the F-test, which tests the goodness of fit of the modeled regression by comparing it to a flat line of the mean of the data. **Table S3A.** Raw retinal values layer thicknesses (in μm) for each age at 1 mm to 7 mm distance from the optic disk rim. **Table S3B.** Percentage of retinal layer thicknesses changes relative to the value at 1 mm from the optic rim of age (raw values in Table S3A). **Table S4A.** Correlation r values between a given retinal layer thicknesses, retinal region and eccentricity from the optic disk rim. **Table S4B.** P-values for correlation r values corresponding to a given retinal layer thickness change, retinal region and eccentricity from the optic disk rim (corresponding to r value in Table S4A). *P*-values for a given correlation coefficient are calculated via the F-test, which tests the goodness of fit of the modeled regression by comparing it to a flat line of the mean of the data.

**Additional file 2: Figure S1.** Comparison of mean (+/− SD) retinal layer thickness with age from the 4 quadrants and the *area centralis*. The dataset for each region is fitted with a linear regression model. **A.** Total retina (TR), **B.** Inner retina (IR), **C.** Inner nuclear layer (INL) and **D.** Ganglion cell complex (GCC). See Additional file 1 - Tables S1A and S1B for raw values and percentage changes with age, respectively, and Additional file 1 - Tables S2A and S2B for r and *p*-values, respectively.

**Additional file 3: Figure S2.** Changes in mean (+/− SD) outer retinal layer thicknesses with age. Receptor+ (REC+), Outer nuclear layer (ONL), and Inner segment/outer segment (IS/OS) changes are shown in this Figure. **A.** Dorsal quadrant, **B.** Ventral quadrant, **C.** Nasal quadrant and **D.** Temporal quadrant. **E.** shows an SD-OCT image of the *area centralis* and **F.** The mean (+/− SD) layer thicknesses in the *area centralis*. See Additional file 1 - Tables S1A and S1B for raw values and percentage changes with age, respectively, and Additional file 1 - Tables S2A and S2B for r and *p*-values, respectively.

**Additional file 4: Figure S3.** Spider graphs of the INL, GCC and IS/OS layer thicknesses in both planes. The mean (+/− SD) layer thickness of the inner nuclear layer (INL; **A** and **B**), ganglion cell complex (GCC; **C** and **D**) and photoreceptor inner segment/outer segment (IS/OS; **E** and **F**) at 4, 6, 12, 26 and 52 weeks of age. **A**, **C** and **E** ventro-dorsal and **B**, **D** and **F** naso-temporal. The INL showed the greatest decrease in thickness with age and a slight decline in thickness with distance from the optic rim. The GCC only thinned slightly with age but thinned markedly with increased distance from the optic rim in all directions. The combined IS/OS changed little with age or distance from optic rim in any direction. A linear mixed effects model was performed to examine the changes in layer thickness with respect to distance from the optic rim (in mm) and age (in weeks). Correlation r values for layer thickness changes with age and distance as well as p-values are shown in Additional file 1 - Tables S4A and 4B, respectively. Additional file 1 - Tables S3A and S3B show raw values and percentage changes with eccentricity.

## Data Availability

Data generated or analysed during this study are included in this published article (and its Additional file information files). The datasets for each individual dog during the current study are available from the corresponding author on reasonable request.

## Abbreviations

AC: *Area centralis*
cSLO: Confocal scanning laser ophthalmoscopy
GCC: Ganglion cell complex
ILM: Inner limiting membrane
INL: Inner nuclear layer
IR: Inner retina
IS: Photoreceptor inner segment
ONL: Outer nuclear layer
OS: Photoreceptor outer segment
REC+: Receptor+
SD-OCT: Spectral Domain – Optical Coherence Tomography
TR: Total retina
