# Supplementary figures and images for: Changes in retinal layer thickness with maturation in the dog: an in vivo spectral domain - optical coherence tomography imaging study
Source: BMC Vet Res. 2020 Jun 30;16:225. doi: 10.1186/s12917-020-02390-8 (PMC7329457; doi:10.1186/s12917-020-02390-8)

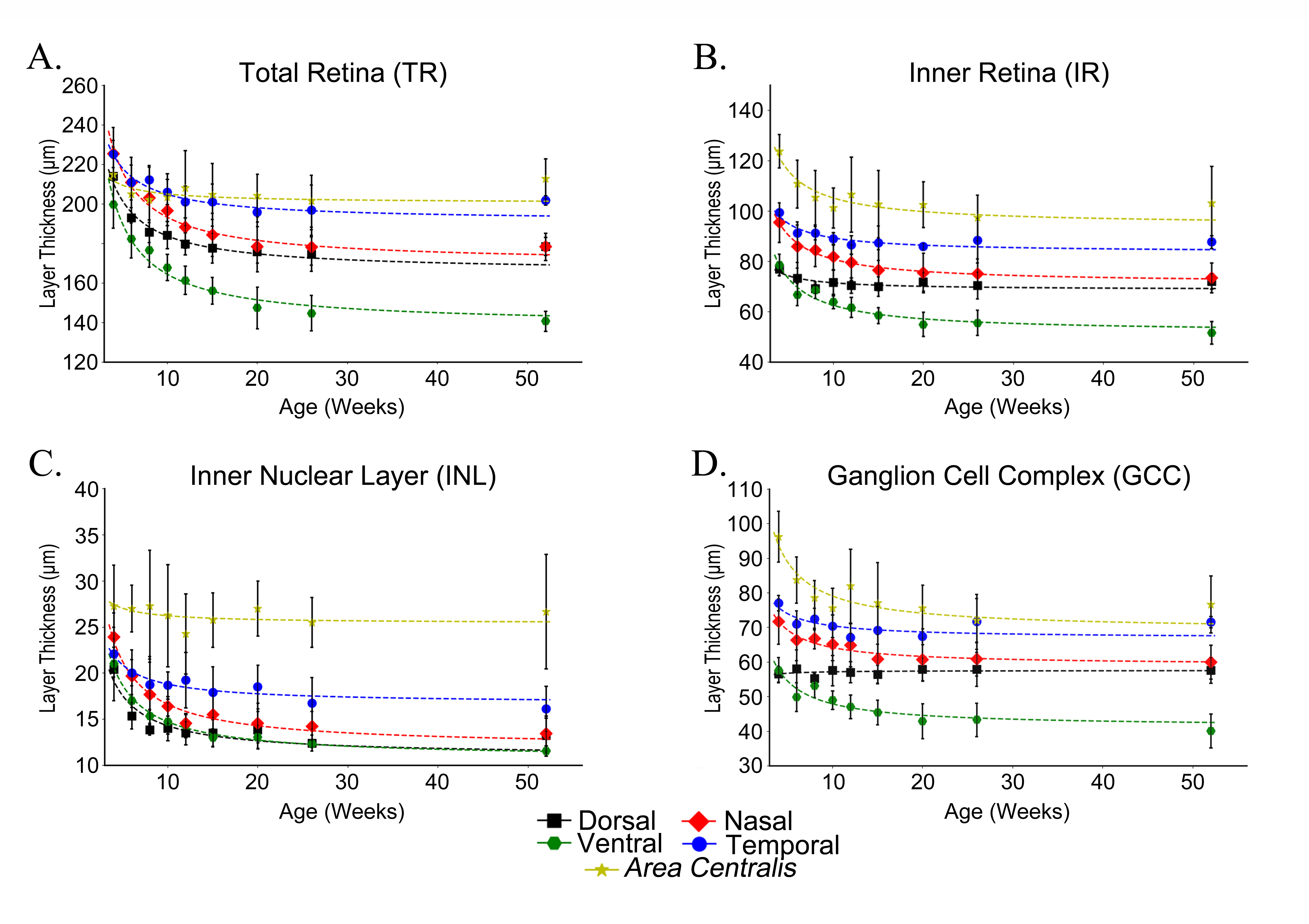

Supplement: Supplementary file 2 — Additional file 2: Figure S1. Comparison of mean (+/− SD) retinal layer thickness with age from the 4 quadrants and the area centralis. The dataset for each region is fitted with a linear regression model. A. Total retina (TR), B. Inner retina (IR), C. Inner nuclear layer (INL) and D. Ganglion cell complex (GCC). See Additional file 1 - Tables S1A and S1B for raw values and percentage changes with age, respectively, and Additional file 1 - Tables S2A and S2B for r and p-values, respectively. [file 12917_2020_2390_MOESM2_ESM.tif]

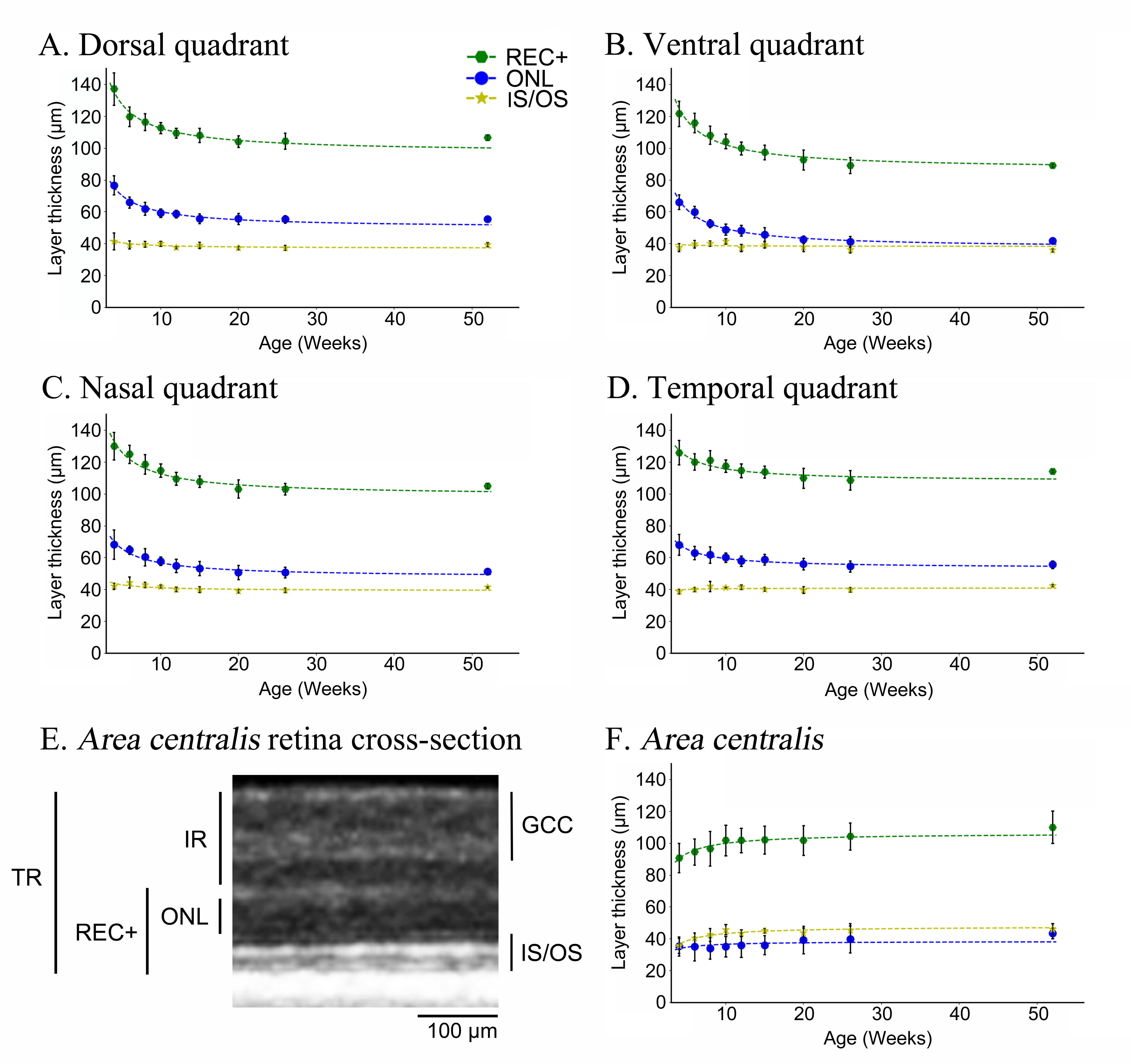

Supplement: Supplementary file 3 — Additional file 3: Figure S2. Changes in mean (+/− SD) outer retinal layer thicknesses with age. Receptor+ (REC+), Outer nuclear layer (ONL), and Inner segment/outer segment (IS/OS) changes are shown in this Figure. A. Dorsal quadrant, B. Ventral quadrant, C. Nasal quadrant and D. Temporal quadrant. E. shows an SD-OCT image of the area centralis and F. The mean (+/− SD) layer thicknesses in the area centralis. See Additional file 1 - Tables S1A and S1B for raw values and percentage changes with age, respectively, and Additional file 1 - Tables S2A and S2B for r and p-values, respectively. [file 12917_2020_2390_MOESM3_ESM.tif]

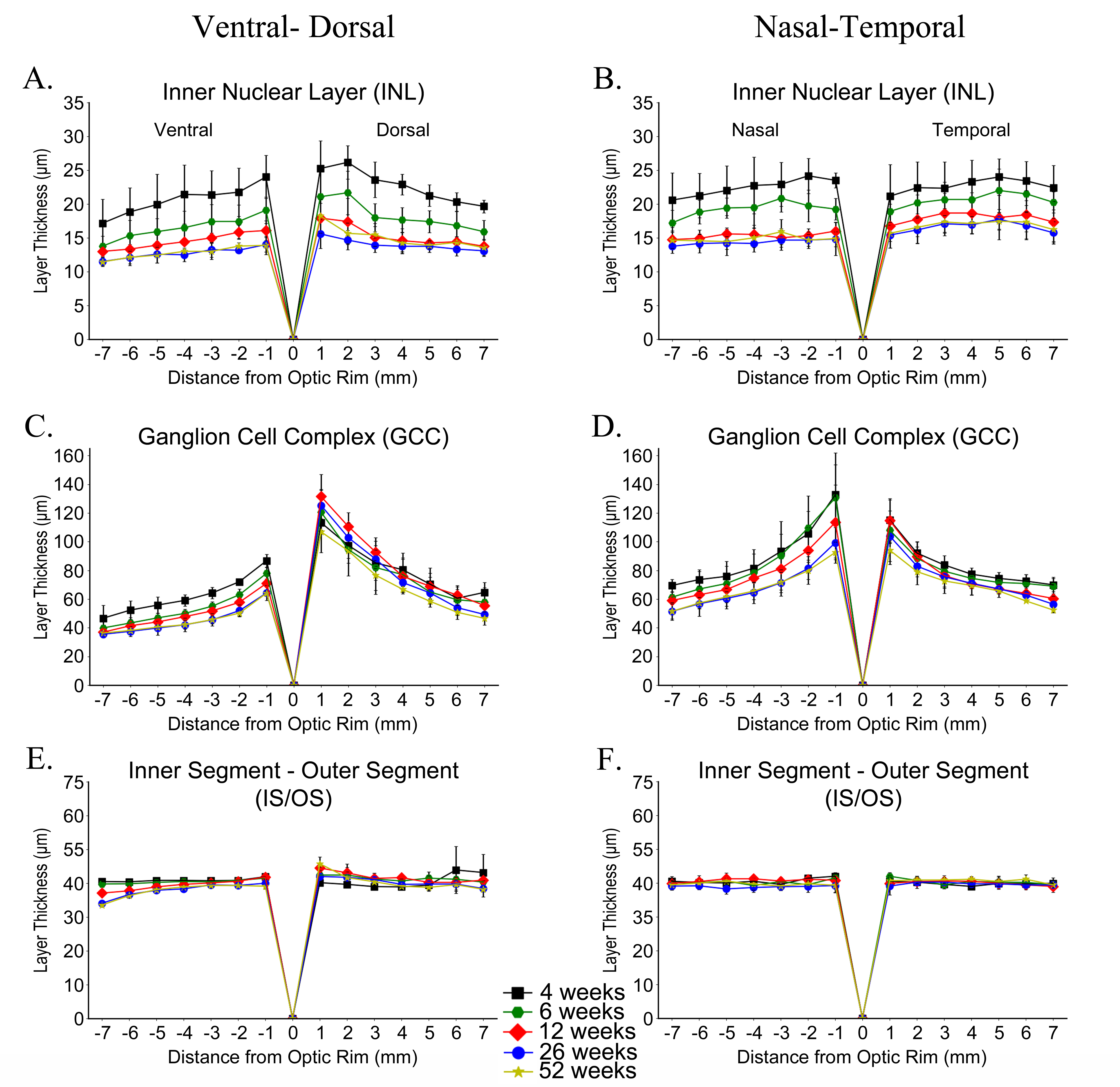

Supplement: Supplementary file 4 — Additional file 4: Figure S3. Spider graphs of the INL, GCC and IS/OS layer thicknesses in both planes. The mean (+/− SD) layer thickness of the inner nuclear layer (INL; A and B), ganglion cell complex (GCC; C and D) and photoreceptor inner segment/outer segment (IS/OS; E and F) at 4, 6, 12, 26 and 52 weeks of age. A, C and E ventro-dorsal and B, D and F naso-temporal. The INL showed the greatest decrease in thickness with age and a slight decline in thickness with distance from the optic rim. The GCC only thinned slightly with age but thinned markedly with increased distance from the optic rim in all directions. The combined IS/OS changed little with age or distance from optic rim in any direction. A linear mixed effects model was performed to examine the changes in layer thickness with respect to distance from the optic rim (in mm) and age (in weeks). Correlation r values for layer thickness changes with age and distance as well as p-values are shown in Additional file 1 - Tables S4A and 4B, respectively. Additional file 1 - Tables S3A and S3B show raw values and percentage changes with eccentricity. [file 12917_2020_2390_MOESM4_ESM.tif]
